# Pulmonary cryptococcosis mimicking metastatic progression despite systemic disease stability in advanced colon cancer: A case report and literature review

**DOI:** 10.1097/MD.0000000000050959

**Published:** 2026-10-02

**Authors:** Yu-cai Jiang, Bi-jin Zheng, Ming-gui Fu, Lin-lin Zheng

**Affiliations:** a Department of Pharmacy, Affiliated Hospital of Putian University, Putian, Fujian Province, China; b School of Pharmacy and Medical Technology, Putian University, Putian, Fujian Province, China; c Department of Oncology, Affiliated Hospital of Putian University, Putian, Fujian Province, China.

**Keywords:** colon cancer, differential diagnosis, multidisciplinary team, pulmonary cryptococcosis, pulmonary nodules

## Abstract

**Rationale:**

In patients with advanced cancer, newly detected pulmonary nodules are often interpreted as metastatic progression. The distinctive feature of this case was the appearance of multiple new lung nodules during otherwise controlled systemic disease: the primary colonic lesion and hepatic metastases remained stable while tumor marker levels declined. This radiologic-clinical discordance prompted reconsideration of metastatic progression and broadened the differential diagnosis.

**Patient concerns:**

A patient with advanced colon cancer, who was undergoing systemic chemotherapy and had otherwise stable disease, was found to have developed new pulmonary nodules on routine surveillance imaging. The patient was entirely asymptomatic from a respiratory standpoint. The primary clinical concern was the discordance between the radiologically suspicious findings and the patient’s overall stable clinical condition.

**Diagnoses:**

The initial radiologic impression favored pulmonary metastases. However, multidisciplinary review recognized the discordance between the new lung lesions and the otherwise stable systemic disease and raised concern for an opportunistic infection. Serum (1,3)-β-D-glucan and qualitative cryptococcal capsular polysaccharide antigen tests were positive, supporting a diagnosis of pulmonary cryptococcosis.

**Interventions:**

After cerebrospinal fluid evaluation excluded central nervous system involvement, fluconazole 400 mg once daily was initiated. The anticancer strategy was not escalated on the basis of the pulmonary findings alone.

**Outcomes:**

Follow-up chest computed tomography demonstrated marked regression of the pulmonary nodules.

**Lessons:**

When new pulmonary lesions arise despite stability or response at established tumor sites, apparent radiologic progression should not automatically be equated with oncologic progression. Integrating the longitudinal treatment response, tumor marker trends, clinical symptoms, imaging features, and targeted infectious testing can prevent unnecessary changes in cancer therapy and may avoid invasive procedures when a sufficiently supported alternative diagnosis is available.

## 1. Introduction

The differential diagnosis of new pulmonary nodules during the follow-up period of systemic treatment for cancer patients is a serious challenge in clinical practice. New lesions on imaging are often initially regarded as disease progression, leading to radical adjustments in treatment strategies.^[1]^ However, sometimes such nodules coexist with well-controlled known lesions, presenting a “contradictory” manifestation, suggesting the possibility of non-tumorous causes such as infection, inflammation, or drug-related lung disease.^[2,3]^ Pulmonary cryptococcosis (PC), as an opportunistic fungal infection, can present as asymptomatic pulmonary nodules in non-HIV-infected patients undergoing chemotherapy, with imaging features highly similar to those of lung metastases, which can easily lead to misdiagnosis.^[4,5]^

Due to the fact that PC and lung metastases both present as nodular shadows on computed tomography (CT) images and lack absolutely specific imaging markers, they are extremely easy to be confused at the initial diagnosis.^[6–8]^ In the follow-up of cancer patients, if PC is misdiagnosed as a metastasis, it will directly affect the treatment decision and prognosis assessment. Therefore, accurate differentiation is of vital importance. In this complex situation, the perspective of a single discipline often has limitations. The multidisciplinary team (MDT) model, by integrating the wisdom of experts from multiple fields such as oncology, respiratory and critical care medicine, radiology, and microbiology, can systematically construct a differential diagnosis framework, significantly improving the accuracy of differentiation and avoiding unnecessary treatment adjustments or delays. This article reports a case of PC mimicking lung metastasis after colon cancer chemotherapy that was successfully identified through MDT, deeply analyzes the differential diagnosis approach, and highlights the key value of MDT in optimizing the diagnosis and treatment process of complex pulmonary lesions.

## 2. Case description

A 67-year-old male patient was diagnosed with a malignant colon tumor (adenocarcinoma of the ascending colon with liver metastasis, T2NxM1 stage IV; KRAS, NRAS, PIK3CA, and BRAF genes were wild-type; microsatellite stable; HER-2 positive). From June 21, 2024, to August 31, 2024, he received chemotherapy with the “bevacizumab + XELOX” regimen for a total of 7 cycles. On September 12, 2024, reexamination of chest and full abdominal CT showed that the local wall thickening of the colon at the hepatic curvature and multiple metastatic tumors in the liver had shrunk compared to before, but the degree of shrinkage was not satisfactory. After full communication with the patient and his family, the treatment plan was adjusted to “bevacizumab + FOLFOXIRI” on September 13, 2024, and 10 cycles of treatment were completed successfully.

On October 24, 2024, the comprehensive assessment showed partial response, and on December 8, 2024, the disease was assessed as stable disease, with continued lesion shrinkage. On January 20, 2025, chest and abdominal CT demonstrated multiple newly developed nodules in the left lung. Given the patient’s stage IV colorectal cancer and known metastatic disease, the appearance of new multifocal pulmonary nodules was initially considered compatible with metastatic spread, making pulmonary metastases a clinically plausible first interpretation. However, this impression was not fully concordant with the overall disease trajectory: the primary colonic lesion and hepatic metastases remained stable, tumor marker levels had decreased, and the patient had no fever, cough, chest tightness, dyspnea, or other respiratory symptoms. This discrepancy between isolated pulmonary radiologic progression and otherwise stable or responding systemic disease created substantial diagnostic uncertainty and prompted consideration of nonmalignant causes, particularly infection. The patient was human immunodeficiency virus (HIV)-negative and reported no history of keeping pets.

Because the nature of the pulmonary nodules remained uncertain, a multidisciplinary consultation was requested on January 22, 2025. The consultation recommended evaluation for pulmonary fungal infection. Serum testing showed positive (1,3)-β-D-glucan and a positive qualitative cryptococcal capsular polysaccharide antigen test, supporting PC. Because the cryptococcal antigen assay used in this case was qualitative rather than titer-based, serial quantitative serum cryptococcal antigen titers were not available for longitudinal assessment. A lumbar puncture was performed on January 26, 2025, and cerebrospinal fluid examination showed no abnormalities, with no evidence of cryptococcal meningitis. Given the positive noninvasive fungal tests, absence of respiratory symptoms, stable general condition, and lack of central nervous system involvement, bronchoscopy and lung biopsy were deferred in favor of antifungal treatment with close clinical and radiologic follow-up. Invasive sampling would have been pursued if the noninvasive work-up had been inconclusive, if the pulmonary lesions enlarged or failed to improve during antifungal therapy, if new respiratory symptoms developed, or if distinguishing infection from metastatic progression remained necessary to guide anticancer treatment. After discussion with the patient and family, fluconazole 400 mg once daily was initiated on January 27, 2025. No clinically significant adverse effects related to fluconazole were observed during treatment. On April 6, 2025, follow-up chest CT showed interval regression of the multiple left lower-lobe nodules. During follow-up to the time of writing, no recurrence of cryptococcal infection was observed (Fig. 1).

**Figure 1.**
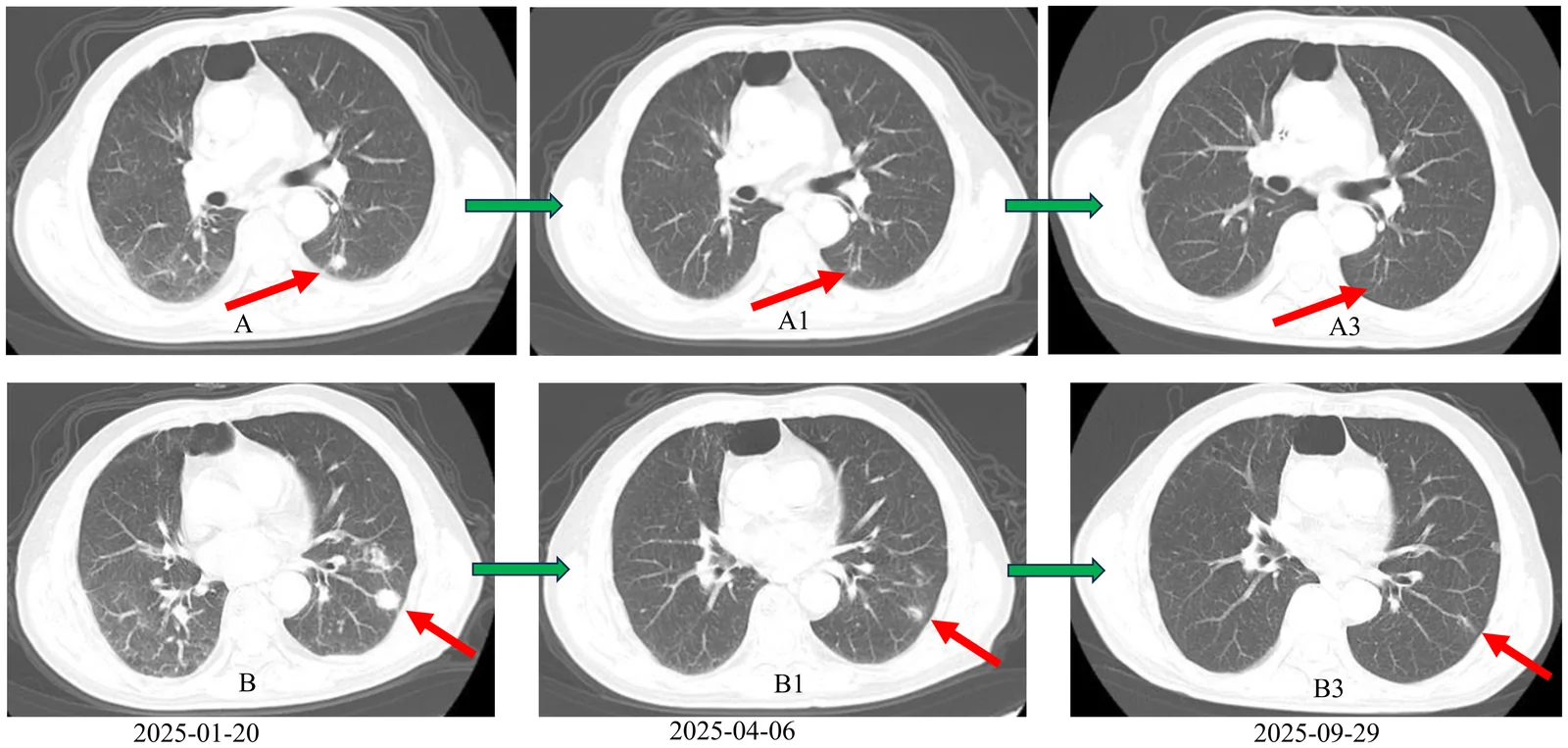
Serial chest CT findings in relation to chemotherapy and antifungal treatment. (A, B) Chest CT obtained on January 20, 2025, after completion of 8 cycles of chemotherapy from September 28, 2024, to January 7, 2025, and before initiation of antifungal therapy, showed newly developed multiple pulmonary nodules in the left lung (red arrows). Fluconazole 400 mg once daily was initiated on January 27, 2025. (A1, B1) Follow-up chest CT on April 6, 2025, approximately 10 weeks after initiation of fluconazole and after 3 additional cycles of chemotherapy administered on February 18, March 4, and March 18, 2025, demonstrated marked regression of the pulmonary nodules. (A3, B3) Follow-up chest CT on September 29, 2025, approximately 8 months after initiation of fluconazole and after 7 additional cycles of chemotherapy administered between April 8 and September 10, 2025, demonstrated further regression, with near-complete resolution of the pulmonary lesions. Red arrows indicate representative pulmonary nodules. CT = computed tomography.

## 3. Discussion

This case illustrates a diagnostic dilemma in a patient with advanced colon cancer who developed new pulmonary nodules during systemic therapy despite otherwise stable disease. The clinically important feature was not simply that PC mimicked metastasis, but that the new lung lesions appeared in direct contrast to the stable primary and hepatic lesions and declining tumor marker levels. This radiologic-clinical discordance became the key signal that triggered reassessment of the initial assumption of metastatic progression.

### 3.1. Radiologic-clinical discordance as a diagnostic signal

The central diagnostic challenge was the separation between the pulmonary imaging findings and the response of established tumor sites. The primary colonic lesion and hepatic metastases had previously achieved partial response and then remained stable, while tumor marker levels decreased; nevertheless, new pulmonary nodules appeared. Although metastatic spread remained plausible because of the patient’s stage IV colorectal cancer, isolated progression in the lungs without parallel worsening elsewhere weakened a purely oncologic explanation. The absence of respiratory symptoms and HIV infection further obscured the possibility of an opportunistic infection. Accordingly, the most informative feature was the longitudinal inconsistency between the new pulmonary abnormalities and the otherwise controlled systemic disease, rather than any single CT characteristic.^[9]^

### 3.2. Multidisciplinary reasoning and a stepwise diagnostic strategy

The multidisciplinary discussion integrated the oncologic treatment trajectory, radiologic findings, pulmonary infection risk, and microbiologic testing into a stepwise diagnostic strategy. Oncology review established that the known colonic and hepatic disease remained controlled; radiology confirmed that the pulmonary nodules represented a new and discordant process; and pulmonology emphasized the possibility of an asymptomatic opportunistic infection in the setting of chemotherapy. This shifted the immediate clinical question from whether anticancer therapy should be intensified to whether the new lesions had a nonmalignant cause. Targeted fungal testing was therefore performed before an irreversible treatment change or invasive lung procedure. The positive (1,3)-β-D-glucan and qualitative serum cryptococcal antigen results, together with exclusion of central nervous system involvement and subsequent radiologic response to fluconazole, supported the final clinical diagnosis.

### 3.3. Comparison with previously reported cancer-associated cases

Selected cancer-associated reports of PC are summarized in Table 1. Most of the verified reports relied on tissue confirmation, but the reasons for invasive diagnosis and the subsequent clinical courses differed substantially. Lee et al^[4]^ described 2 asymptomatic patients with gynecologic cancers in whom new pulmonary nodules were presumed to represent metastases. Both underwent thoracoscopic lobectomy; serum cryptococcal antigen testing was negative in both cases, and histopathology established PC. After fluconazole treatment, the patients were disease-free at 12 and 13 months, respectively. In the report by Wang et al^[10]^, 2 pulmonary lesions initially appeared to represent a primary lung cancer with intrapulmonary metastasis. Bronchoscopy was nondiagnostic, and separate CT-guided biopsies demonstrated lung adenocarcinoma in 1 lesion and PC in the other; surgery plus 6 months of fluconazole was followed by no recurrence of either disease over 5 years. Yao et al^[13]^ similarly showed the importance of lesion-specific assessment: multiple heterogeneous lung lesions were initially concerning for metastatic disease, but CT-guided biopsies of 3 lesions identified bilateral primary lung adenocarcinoma together with PC. After local treatment, rituximab, cyclophosphamide, doxorubicin, vincristine, and prednisone, and fluconazole, no tumor recurrence, progression, or metastasis was reported during 27 months of follow-up.

**Table 1:** Selected reports of pulmonary cryptococcosis in patients with cancer: diagnostic pathways and clinical outcomes.

Year	Author	Underlying malignancy/treatment background	Presentation	Initial diagnostic concern	Diagnostic pathway	Clinical outcome
2023	Lee et al^[4]^	Cervical cancer after chemoradiotherapy; ovarian cancer after chemotherapy	Asymptomatic; tumor markers not elevated	Pulmonary metastases	PET/CT → thoracoscopic lobectomy; serum CrAg negative in both cases	Fluconazole; disease-free at 12 and 13 mo, respectively
2024	Wang et al^[10]^	Synchronous lung adenocarcinoma and pulmonary cryptococcosis	Asymptomatic	Primary lung cancer with suspected intrapulmonary metastasis	Bronchoscopy nondiagnostic → separate CT-guided biopsies of 2 nodules	Lung cancer surgery + fluconazole for 6 mo; no recurrence of either disease at 5 yr
2016	Sakamoto and Hisaoka^[11]^	Ewing sarcoma after intensive multiagent chemotherapy	Incidental 5-mm nodule at completion of chemotherapy	Pulmonary metastasis	Surgical resection of the pulmonary nodule	Fluconazole for 18 wk; no recurrence at 2 yr
2023	Kobe et al^[12]^	Recurrent EGFR-mutant lung adenocarcinoma on erlotinib + ramucirumab	Asymptomatic	Disease progression; rebiopsy for EGFR T790M	Percutaneous needle biopsy; serum/CSF CrAg negative	Fluconazole; pulmonary lesion shrank while liver metastases enlarged
2024	Yao et al^[13]^	Gastric DLBCL with synchronous bilateral lung adenocarcinoma and pulmonary cryptococcosis	Hematemesis; pulmonary lesions detected on staging CT	Metastatic disease; differential also included primary lung tumors and infection	CT-guided biopsies of 3 pulmonary lesions	Local lung treatment + R-CHOP + fluconazole; no tumor recurrence/progression/metastasis at 27 mo
2024	Epstein et al^[14]^	Relapsed/refractory multiple myeloma after multiple lines of therapy	Bilateral consolidations; later cough and sore throat	Infectious, inflammatory, or neoplastic pulmonary process	Serum CrAg 1:256; CSF negative; bronchoscopy/biopsy deferred because of frailty	Fluconazole; symptoms and CT findings improved; myeloma therapy resumed
2025	Hamada et al^[15]^	EGFR L858R lung adenocarcinoma on osimertinib + dexamethasone	Asymptomatic	Disease progression vs secondary infection	Early CT-guided biopsy; serum CrAg negative; β-D-glucan markedly elevated	Liposomal amphotericin B + flucytosine → fluconazole; radiologic regression
2022	Eremiev et al^[16]^	Rectal adenocarcinoma after neoadjuvant FOLFOX-panitumumab, surgery, and completion of chemotherapy	Pulmonary opacities detected on surveillance CT	Indeterminate hypermetabolic pulmonary opacities; malignancy remained in the differential	Empirical antibiotics → PET/CT → bronchoscopy/BAS/BAL nondiagnostic → CT-guided biopsy showing fungal spores/no malignancy → serum CrAg 1:80	Fluconazole for 6 mo; pulmonary lesions decreased on follow-up CT

BAS = bronchial aspirate, BAL = bronchoalveolar lavage, CSF = cerebrospinal fluid, CT = computed tomography, DLBCL = diffuse large B-cell lymphoma, EGFR = epidermal growth factor receptor, PET = positron emission tomography, R-CHOP = rituximab, cyclophosphamide, doxorubicin, vincristine, and prednisone.

Other reports illustrate how the diagnostic pathway should be adapted to the clinical context. Kobe et al^[12]^ reported an asymptomatic patient with recurrent epidermal growth factor receptor-mutant lung adenocarcinoma treated with erlotinib plus ramucirumab who developed a new pulmonary lesion together with liver lesions. Percutaneous rebiopsy, performed to assess epidermal growth factor receptor T790M, unexpectedly demonstrated PC; serum and cerebrospinal fluid cryptococcal antigens were negative. After fluconazole, the pulmonary lesion shrank while the liver metastases enlarged, directly demonstrating discordant infectious and malignant disease trajectories. Hamada et al^[15]^ described an asymptomatic patient receiving osimertinib and dexamethasone whose new pulmonary lesions raised concern for disease progression versus secondary infection. CT-guided biopsy was performed early and confirmed PC; serum cryptococcal antigen was negative while serum β-D-glucan was markedly elevated. Liposomal amphotericin B plus flucytosine followed by fluconazole produced radiologic regression. In contrast, Epstein et al^[14]^ reported a patient with multiple myeloma whose bilateral pulmonary consolidations were considered infectious, inflammatory, or neoplastic. When serum cryptococcal antigen later became positive at 1:256, bronchoscopy or lung biopsy was considered but deferred because of frailty; fluconazole led to symptomatic and radiologic improvement, allowing myeloma therapy to resume. Sakamoto and Hisaoka^[11]^ reported a 5-mm pulmonary nodule detected at completion of intensive chemotherapy for Ewing sarcoma. Because pulmonary metastasis was strongly suspected, the nodule was surgically resected and was found to be PC; fluconazole for 18 weeks was followed by no recurrence during 2 years of follow-up. Eremiev et al^[16]^ described a patient with rectal adenocarcinoma who developed indeterminate right lower-lobe pulmonary opacities during posttreatment surveillance. After empirical antibiotics, the opacities enlarged and remained hypermetabolic on positron emission tomography/CT. Bronchoscopy with bronchial aspirate and bronchoalveolar lavage was nondiagnostic, while CT-guided needle biopsy showed parenchymal necrosis with fungal spores and no malignancy; serum cryptococcal antigen was positive at 1:80. A 6-month course of fluconazole was followed by radiologic reduction of the lesions.

Taken together, the verified reports show several complementary diagnostic patterns. Invasive tissue confirmation was commonly used when metastatic progression or a second malignancy remained plausible, particularly when serum cryptococcal antigen testing was negative or when different pulmonary lesions could represent different diseases. A noninvasive treatment-response strategy was used selectively when cryptococcal antigen testing supported the diagnosis and invasive procedures posed substantial clinical burden. Eremiev et al^[16]^ illustrate an intermediate, sequential approach in which empirical antibiotics, positron emission tomography/CT, bronchoscopy with lavage, CT-guided biopsy, serum cryptococcal antigen testing, and subsequent treatment response were integrated after earlier tests were nondiagnostic. The present case differs in that new pulmonary nodules arose while the known colonic and hepatic disease remained stable and tumor marker levels declined, and positive noninvasive fungal tests allowed antifungal treatment and close radiologic surveillance without immediate bronchoscopy or lung biopsy. The subsequent regression of the pulmonary nodules supported the diagnosis, although the absence of histopathologic confirmation remains an important limitation.

### 3.4. Strengths and limitations

The key strengths of this case are the longitudinal demonstration of discordance between new pulmonary lesions and stable or responding disease at established tumor sites; the explicit integration of oncologic, radiologic, pulmonary, and microbiologic information into a stepwise diagnostic process; and the availability of serial imaging showing regression after targeted antifungal therapy. These features provide a clear clinical reasoning narrative and show how premature reclassification as metastatic progression can be avoided. The main limitations are the absence of histopathologic or culture confirmation from lung tissue and the lack of serial quantitative serum cryptococcal antigen titers. Accordingly, the diagnosis should be interpreted as a clinically well-supported diagnosis rather than a pathologically proven one. As a single case report, this study also cannot establish the general effectiveness of multidisciplinary management or quantify its effect on misdiagnosis rates.

## 4. Conclusion

This case illustrates that new pulmonary nodules arising during cancer therapy should be interpreted in the context of the patient’s overall disease trajectory rather than as automatic evidence of metastatic progression. When pulmonary findings are discordant with stable or responding known disease, opportunistic infections such as PC should be considered. Multidisciplinary review and targeted noninvasive testing may help avoid premature changes in anticancer therapy and, in selected clinically stable patients, may allow invasive procedures to be deferred while close radiologic follow-up is maintained.

## Author contributions

**Conceptualization:** Lin-lin Zheng, Yu-cai Jiang, Bi-jin Zheng, Ming-gui Fu.

**Data curation:** Lin-lin Zheng, Yu-cai Jiang, Bi-jin Zheng.

**Formal analysis:** Lin-lin Zheng, Yu-cai Jiang.

**Funding acquisition:** Lin-lin Zheng.

**Investigation:** Lin-lin Zheng, Yu-cai Jiang, Bi-jin Zheng.

**Methodology:** Yu-cai Jiang.

**Validation:** Bi-jin Zheng.

**Visualization:** Lin-lin Zheng.

**Writing – original draft:** Yu-cai Jiang.

**Writing – review & editing:** Lin-lin Zheng, Yu-cai Jiang, Bi-jin Zheng, Ming-gui Fu.
